# Multicenter prospective blinded melanoma detection study with a handheld elastic scattering spectroscopy device

**DOI:** 10.1016/j.jdin.2023.10.011

**Published:** 2023-11-15

**Authors:** Rebecca I. Hartman, Nicole Trepanowski, Michael S. Chang, Kelly Tepedino, Christopher Gianacas, Jennifer M. McNiff, Maxwell Fung, Naiara Fraga Braghiroli, Jane M. Grant-Kels

**Affiliations:** a Department of Dermatology, Brigham and Women’s Hospital, Boston, Massachusetts; b Harvard Medical School, Boston, Massachusetts; c Department of Dermatology, VA Integrated Service Network (VISN-1), Jamaica Plain, Massachusetts; d Boston University School of Medicine, Boston, Massachusetts; e North Florida Dermatology, Lake City, Florida; f The George Institute for Global Health, UNSW Sydney, Sydney, Australia; g School of Population Health, UNSW Sydney, Sydney, Australia; h Departments of Dermatology and Pathology, Yale School of Medicine, New Haven, Connecticut; i University of California Davis School of Medicine, Sacramento, California; j Dermatology Department, Miami Cancer Institute, Miami, Florida; k Department of Dermatology, University of Connecticut School of Medicine, Farmington, Connecticut; l Department of Dermatology, University of Florida College of Medicine, Gainesville, Florida

**Keywords:** AI, artificial intelligence, automated, biopsy, DermaSensor, DERM-ASSESS III, detection, elastic scattering device, elastic scattering spectroscopy, ESS, handheld, melanoma, non-invasive, NPV, pigmented lesion, PPV, sensitivity, skin cancer, specificity, spectroscopic, spectroscopy, technology

## Abstract

**Background:**

The elastic scattering spectroscopy (ESS) device (DermaSensor Inc., Miami, FL) is a noninvasive, painless, adjunctive tool for skin cancer detection.

**Objectives:**

To investigate the performance of the ESS device in the detection of melanoma.

**Methods:**

A prospective, investigator-blinded, multicenter study was conducted at 8 United States (US) and 2 Australian sites. All eligible skin lesions were clinically concerning for melanoma, examined with the ESS device, subsequently biopsied according to dermatologists’ standard of care, and evaluated with histopathology. A total of 311 participants with 440 lesions were enrolled, including 44 melanomas (63.6% in situ and 36.4% invasive) and 44 severely dysplastic nevi.

**Results:**

The observed sensitivity of the ESS device for melanoma detection was 95.5% (95% CI, 84.5% to 98.8%, 42 of 44 melanomas), and the observed specificity was 32.5% (95% CI, 27.2% to 38.3%). The positive and negative predictive values were 16.0% and 98.1%, respectively.

**Limitations:**

The device was tested in a high-risk population with lesions selected for biopsy based on clinical and dermoscopic assessments of board-certified dermatologists. Most enrolled lesions were pigmented.

**Conclusion:**

The ESS device’s high sensitivity and NPV for the detection of melanoma suggest the device may be a useful adjunctive, point-of-care tool for melanoma detection.

**Capsule Summary**

• Novel technologies for melanoma detection may improve diagnostic performance, increase access, reduce resource utilization, and decrease healthcare costs.
• In this study, the high sensitivity and negative predictive value of a noninvasive and easy-to-use elastic scattering spectroscopy device suggest that this point-of-care tool may be a useful adjunct for melanoma detection.

## Introduction

Melanoma incidence has increased faster than nearly all other cancers.^1^ Although representing less than 5% of cutaneous malignancies, melanoma accounts for most skin cancer deaths.^1^ Novel therapies approved since 2011 have improved survival for late-stage melanomas but are costly and have significant side effects.^2^^,^^3^

Dermatologists utilize tools to evaluate lesions for melanoma including dermoscopy, confocal microscopy, longitudinal photography and digital dermoscopy, gene expression testing, and artificial intelligence (AI).^4^ Use of these technologies varies by training and access. The number of skin lesions needed to biopsy (NNB) to diagnose 1 melanoma varies from 14.8 for clinicians, 7.5 for dermatologists, and 13.2 for US-based dermatologic practitioners (including dermatologists and advanced practice professionals).^5^ Biopsied benign lesions increase morbidity and healthcare costs.

A handheld device (DermaSensor, DermaSensor Inc) using elastic scattering spectroscopy (ESS) and AI has been developed as an adjunctive tool for skin cancer detection.^6^^,^^7^ ESS utilizes the spectral recording of photons scattered back from refractive-index gradients to gain information about cellular and sub-cellular structures.^8^ Different tissue types and histopathologic changes, such as malignancy, exhibit specific optical signatures.^8^ In the primary care setting, the ESS device has a sensitivity of 90.0% to 95.5% and a specificity of 20.7% to 60.7% for detecting melanoma and keratinocyte carcinomas.^6^^,^^7^ Our objective was to investigate the real-world performance of the ESS device for melanoma detection using skin lesions identified as potential melanomas by board-certified dermatologists.

## Methods

### Ethical conduct

The study was approved by a central institutional review board (IRB) and local IRBs or ethics committees and was conducted in accordance with the revised Declaration of Helsinki, Good Clinical Practice guidelines, national and data protection laws, and applicable regulatory requirements. The study sponsor developed the trial protocol, provided ESS devices and funding to carry out the study, and engaged a contract research organization (CRO) to oversee study operations and a biostatistics firm to conduct data analyses. As the study was noninterventional, clinical trial registration was not required.

### Study design and data acquisition

The DERMaSensor Use in the ASSESSment of Skin Lesions Suggestive of Melanoma III (DERM-ASSESS III) was a blinded, prospective study performed at 8 sites in the US and 2 sites in Australia from December 2020 through October 2021. One site was an academic institution (Brigham and Women’s Hospital/Dana Farber Cancer Institute); the remaining 9 sites were high melanoma volume private practice dermatology clinics. All 12 enrolling dermatologist-investigators were board-certified. Potential participants were screened according to inclusion and exclusion criteria (Supplementary Table I, available via Mendeley at https://data.mendeley.com/datasets/fcgghmx8f4/1). All participants provided written informed consent, and participation did not impact treatment. Electronic case records recorded participant demographics and melanoma risk factors. All enrolled lesions were clinically suspicious for melanoma. Dermatologist-investigators performed dermoscopy assessments per their standard of care and were asked to clinically predict histopathologic diagnoses as benign, melanoma, or severely dysplastic nevus (SDN).

Dermatologist-investigators and the sponsor were blinded to device output. A CRO blinded case records, ESS device measurements, and histopathologic reports from the sponsor.

### Histopathologic evaluation as the reference standard

Biopsies were performed using dermatologist-investigators’ standard of care and were assessed with dermatopathology consensus review via a primary and secondary consensus process. Each lesion was independently assessed by a local board-certified dermatopathologist and a subsequent dermatopathology over-read by a board-certified dermatopathologist was performed (primary consensus). For lesions diagnosed as benign during primary consensus, no further review was required. For lesions diagnosed as melanoma, SDN, moderately DN, or other potentially high-risk or inconclusive diagnoses during primary consensus, further blinded review was conducted by 2 or 3 additional expert dermatopathologists (secondary consensus). The histopathologic reference standard refers to the diagnosis after final consensus was achieved. Only lesions with histopathologic reference standard diagnoses were included in the analysis population.

### ESS Device

The ESS device is a noninvasive, point-and-click spectrometer system weighing 1.9 kg in total, with the wireless handheld piece weighing 0.3 kg (Supplementary Fig 1, available via Mendeley at https://data.mendeley.com/datasets/fcgghmx8f4/1).^9^ The unit assesses skin lesions ≥ 2.5 mm in diameter in less than 30 seconds. The device emits light pulses of hundreds of distinct wavelengths and receives and analyzes spectral data from cellular and sub-cellular structures. The unit captures 5 recordings to generate a spectral reading which is analyzed by a locked algorithm in the device’s software. The volume of tissue that the device evaluates with each spectral recording was estimated computationally with Monte Carlo simulations to approximately 0.7 mm (length) × 0.4 mm (width) × 0.5 mm (depth).^10^ The device classifies lesions as “Investigate Further” or “Monitor,” with the “Investigate Further” results accompanied by a spectral score of 1 to 10, with 10 being most similar to previously validated malignant lesions.^7^ Lesions classified as “Monitor” do not receive a spectral score. Lesions classified as “Investigate Further” by the ESS device are interpreted as high risk, that is high risk of malignancy, whereas “Monitor” results are interpreted as low risk, that is low risk of malignancy.^8^

### Outcome measures

Primary endpoints were device performance for distinguishing histopathologically benign lesions from those histopathologically diagnosed as melanoma (excluding other malignant diagnoses, eg, keratinocyte carcinomas and SDN) and the combined outcome of melanoma and SDN.

Statistical analyses included concordance analyses (sensitivity, specificity, positive predictive value (PPV), and negative predictive value (NPV)) using dermatopathology with clustered confidence intervals (CIs) based on Wilson score and subgroup analyses based on participant and lesion clinical characteristics.^11^ Area under the receiver operating characteristic curve (AUROC) statistics for device and dermatologist-investigators were used for comparative effectiveness analyses. PPV analyses were conducted to assess device performance across various spectral score groupings, and Wald test *P*-values were presented. NNB for melanoma detection was calculated using the inverse of the PPV.^12^ *P* < .05 and nonoverlapping CIs were considered statistically significant. Analyses were performed using SAS 9.4 (SAS Institute), R version 4.2 (R Core Team), and STATA 17 (StataCorp).

## Results

Of 332 recruited subjects, the final study population contained 311 subjects with 440 lesions suspicious for melanoma (Supplementary Fig 2, available via Mendeley at https://data.mendeley.com/datasets/fcgghmx8f4/1). The average participant was 62.0 ± 15.4 years-old, and participants included 53.7% males and 46.3% females (Table I). Most participants identified as non-Hispanic (96.1%) and white (97.7%). Participants had an average of 1.4 ± 0.8 lesions enrolled.

**Table I:** Participant characteristics

Characteristics	Participants with eligible lesions *n* = 311, *n* (%)
Age - years, Mean (SD)	62.0 (15.4)
Sex	
Male	167 (53.7)
Female	144 (46.3)
Ethnicity	
Hispanic or Latino	8 (2.6)
Not Hispanic or Latino	299 (96.1)
Unknown	4 (1.3)
Race	
White	304 (97.7)
Other	3 (1.0)
Native Hawaiian or other Pacific Islander	2 (0.6)
White, Native Hawaiian or other Pacific Islander	1 (0.3)
Asian	1 (0.3)
Fitzpatrick skin type∗	
I - Always burns, never tans	32 (10.3)
II - Always burns, tans minimally	165 (53.1)
III - Sometimes mild burn, tans uniformly	66 (21.2)
IV - Burns minimally, always tans well	20 (6.4)
V - Very rarely burns, tans very easily	20 (6.4)
VI - Never burns, never tans	8 (2.6)
Melanoma risk factors†	
Ultraviolet light exposure (natural or tanning bed)	143 (46.0)
Personal history of skin cancer	119 (38.3)
Fair skin, freckling, light hair	107 (34.4)
Family history of skin cancer	70 (22.5)
Many moles and/or dysplastic nevi	58 (18.6)
New or changing lesion(s)	41 (13.2)
Weakened immune system	8 (2.6)
No risk factors reported	57 (18.3)
Person who discovered the lesion(s) of concern	
Health care provider	224 (72.0)
Patient	80 (25.7)
Family member/partner	7 (2.3)

*SD*, Standard deviation.

∗ Determined by clinical judgment or assessment.

† Percentages do not sum to 100% because participants may have had more than 1 melanoma risk factor.

Lesions were located on the trunk (61.6%), head and neck (15.5%), upper extremity (13.0%), and lower extremity (10.0%) (Table II). The average lesion size was 5.8 mm × 4.7 mm. Lesions were predominantly flat (83.6%), smooth (91.1%), and pigmented (96.8%). Histopathologic diagnoses included benign (74.1%), SDN (10.0%), melanoma (10.0%), or malignant other (5.9%, basal cell carcinoma, squamous cell carcinoma, and atypical fibroxanthoma) (Table III). Overall study cancer prevalence was 15.9%, and NNB for melanoma detection by dermatologist-investigators was 10.

**Table II:** Lesion characteristics

Characteristics	*n* = 440, *n* (%)
Anatomic location	
Trunk	271 (61.6)
Head and neck	68 (15.5)
Upper extremity	57 (13.0)
Lower extremity	44 (10.0)
Length (mm), mean (SD)	5.8 (2.7)
Width (mm), mean (SD)	4.7 (2.0)
Flat or elevated	
Flat	368 (83.6)
Elevated	72 (16.4)
Texture	
Smooth	401 (91.1)
Rough	39 (8.9)
Pigmentation	
Pigmented	426 (96.8)
Non-pigmented	14 (3.2)

*mm*, Millimeter; *SD*, standard deviation.

**Table III:** Clinical and histopathologic diagnoses of lesions

Characteristics	*n* = 440, *n* (%)
Dermatologist-investigator prediction	
Benign	224 (50.9)
Severely dysplastic nevus	128 (29.1)
Melanoma	88 (20.0)
Dermatologist-investigator level of confidence	
High	319 (72.5)
Low	121 (27.5)
Histopathologic diagnosis	
Benign, *n* = 326	
Benign nevus	108 (24.5)
Mildly dysplastic nevus	88 (20.0)
Seborrheic keratosis	57 (13.0)
Solar lentigo	22 (5.0)
Actinic keratosis	12 (2.7)
Benign - discordant∗	10 (2.3)
Moderately dysplastic nevus	8 (1.8)
Lichenoid keratosis	6 (1.4)
Benign other†	5 (1.1)
Simple lentigo	4 (0.9)
Blue nevus	3 (0.7)
Verruca	1 (0.2)
Epidermal cyst	1 (0.2)
Dermatofibroma	1 (0.2)
Severely dysplastic nevus, *n* = 44	44 (10.0)
Atypical melanocytic hyperplasia	30 (68.2)
Atypical melanocytic proliferation	14 (31.8)
Malignant, *n* = 70	
Melanoma	44 (10.0)
Thickness, *n* = 44	
In situ	28 (63.6)
≤1.0 mm	12 (27.3)
>1.0-2.0 mm	1 (2.3)
>2.0-4.0 mm	2 (4.5)
>4.0 mm	1 (2.3)
Basal cell carcinoma	13 (3.0)
Squamous cell carcinoma	12 (2.7)
Malignant other‡	1 (0.2)

*mm*, Millimeter.

∗ Benign-discordant lesions were consistently considered benign/low risk histopathologic diagnoses during review by the expert dermatopathologists but received different benign diagnoses during primary review and secondary review processes.

† Benign other diagnoses include poroma, post-inflammatory pigmentary alternation, inflamed verrucoid keratosis, ochronosis, and foreign body reaction.

‡ Other malignant diagnosis was atypical fibroxanthoma (malignant fibrous histiocytoma).

The dichotomous outcome of the ESS device was compared with the histopathologic reference standard. Sensitivity of the ESS device for detecting melanoma was 95.5% (95% CI, 84.5% to 98.8%, 42 of 44 melanomas) (Table IV). Specificity of the ESS device was 32.5% (95% CI, 27.2% to 38.3%), NPV was 98.1% (95% CI, 91.8% to 99.6%), and PPV was 16.0% (95% CI, 11.6% to 21.7%). The 2 device false negative melanomas were a 10 mm × 6 mm flat, smooth, and pigmented lesion on the trunk that was histopathologically diagnosed as a “Level 2 superficial spreading melanoma with 0.3 mm thickness” (Supplementary Fig 3, available via Mendeley at https://data.mendeley.com/datasets/fcgghmx8f4/1) and a 9 mm × 4.6 mm flat, smooth, and pigmented lesion on the lower extremity that was histopathologically diagnosed as a “Level 1 superficial spreading melanoma in situ” (Supplementary Fig 4, available via Mendeley at https://data.mendeley.com/datasets/fcgghmx8f4/1).

**Table IV:** Concordance between ESS device∗ and histopathologic reference standard† for detection of melanoma

Histopathologic diagnosis†	ESS Device∗	
	High risk	low risk
Melanoma, *n* = 44, *n* (%)	42 (95.5)	2 (4.5)
Benign, *n* = 326, *n* (%)	220 (67.5)	106 (32.5)
Specificity (95% CI‡)	0.325 (0.272-0.383)	
Sensitivity (95% CI‡)	0.955 (0.845-0.988)	
NPV (95% CI‡)	0.981 (0.918-0.996)	
PPV (95% CI‡)	0.160 (0.116-0.217)	

*CI*, Confidence interval; *ESS*, elastic scattering spectroscopy; *NPV*, negative predictive value; *PPV*, positive predictive value.

∗ Lesions that were classified by the ESS device as “Investigate Further” were interpreted as high risk, whereas lesions classified as “Monitor” were interpreted as low risk.

† Severely dysplastic nevus (*n* = 44) and other malignant diagnoses [(basal cell carcinoma (*n* = 13), squamous cell carcinoma (*n* = 12), and atypical fibroxanthoma (*n* = 1)] are excluded from this table.

‡ 95% CIs were calculated accounting for the within-subject correlation using the Wilson method.

Comparatively, device performance for detecting melanoma and SDN yielded a sensitivity of 90.9% (95% CI, 83.1% to 95.3%), specificity of 32.5% (95% CI, 27.2% to 38.3%), NPV of 93.0% (95% CI, 85.7% to 96.7%), and PPV of 26.7% (95% CI, 21.5% to 32.6%) (Supplementary Table II, available via Mendeley at https://data.mendeley.com/datasets/fcgghmx8f4/1). The device yielded similar sensitivities with overlapping CIs for lesions that were clinically suspicious for melanoma but were histopathologically diagnosed as other malignant diagnoses (Supplementary Table III and IV, available via Mendeley at https://data.mendeley.com/datasets/fcgghmx8f4/1).

Specificity of the device was higher for lesions <6 mm (39.3%, 95% CI, 32.2% to 46.8%) than for lesions ≥6 mm (19.6%, 95% CI, 13.2% to 28.2%) (Supplementary Figs 5 and 6, available via Mendeley at https://data.mendeley.com/datasets/fcgghmx8f4/1). Sensitivity of the device by lesion size could not be compared due to study sample size.

The PPV for melanoma detection for lesions with spectral scores of 6-10 was 31.9% (95% CI, 20.2% to 46.4%), compared to 12.6% (95% CI, 8.4% to 18.4%) for lesions with spectral scores of 1-5 (*P* < .001, Table V). The NNB to detect melanoma was 3.1 and 7.9 for spectral scores of 6-10 and 1-5, respectively. Spectral scores of 8-10 yielded a PPV of 47.4% (95% CI, 24.9% to 69.8%) (*P* < .001, scores 8-10 vs scores 4-7), scores of 4-7 yielded a PPV of 20.5% (95% CI, 12.7% to 31.5%) (*P* = .003, scores 4-7 vs scores 1-3), and scores of 1-3 yielded a PPV of 10.3% (95% CI, 6.3% to 16.5%). For spectral scores of 8-10, 4-7, and 1-3, the NNB to detect melanoma was 2.1, 4.9, and 9.7, respectively. Findings were similar for the combined outcome of melanoma and SDN (Supplementary Table V, available via Mendeley at https://data.mendeley.com/datasets/fcgghmx8f4/1). Of the SDN classified by the device as high risk (86.4%), 94.7% had a spectral score of 1-5.

**Table V:** Spectral score∗ breakdown for detection of melanoma in lesions classified by the ESS device as high risk

Spectral score∗	Benign†, *n* = 220, *n* (%)	Melanoma†, *n* = 42, *n* (%)	PPV (95% CI)‡	NNB	*P*-value§
Group 1					
1-5	188 (85.5)	27 (64.3)	0.126 (0.084-0.184)	7.9	
6-10	32 (14.5)	15 (35.7)	0.319 (0.202-0.464)	3.1	<.001
Group 2					
1-3	148 (67.3)	17 (40.5)	0.103 (0.063-0.165)	9.7	
4-7	62 (28.2)	16 (38.1)	0.205 (0.127-0.315)	4.9	.003‖
8-10	10 (4.5)	9 (21.4)	0.474 (0.249-0.698)¶	2.1	<.001#

*CI*, Confidence interval; *NNB*, number needed to biopsy; *PPV*, positive predictive value.

∗ Lesions that were classified by the ESS device as “Investigate Further” were interpreted as high risk, whereas lesions classified as “Monitor” were interpreted as low risk. “Investigate Further” results are accompanied by a spectral score of 1 to 10, with 10 being most similar to previously validated malignant lesions.

† Histopathologic diagnoses.

‡ The PPV calculation only considers lesions classified by the device as high risk. 95% CIs were calculated accounting for the within-subject correlation using the Wilson method.

§ The Wald method was used to compare PPVs.

‖ The *P*-value compares PPVs for lesions with spectral scores of 1-3 vs 4-7.

¶ Where the sample size was too small to utilize the Wilson method for calculating CIs, the Wald method was used.

# The *P*-value compares PPVs for lesions with spectral scores of 4-7 vs 8-10.

AUROC comparisons for clinical predictions by the device versus dermatologist-investigators yielded similar results for the detection of melanoma (AUROC 0.758 vs 0.747; *P* = .829) and for melanoma and SDN (AUROC 0.652 vs 0.633; *P* = .700, Supplementary Table VI, available via Mendeley at https://data.mendeley.com/datasets/fcgghmx8f4/1).

## Discussion

The ESS device was highly sensitive with a high NPV for detecting melanoma and the combined outcome of melanoma and SDN. In real-world use, a negative ESS device result may be useful in reducing healthcare resource utilization and morbidity. The previously reported sensitivity for melanoma detection by referral centers/experts with clinical examination (69%) and dermoscopy (87%) is similar to the sensitivity of the ESS device for melanoma detection in this study (95.5%), although the values are not directly comparable due to differences in study designs and populations.^13^ The specificity of the ESS device for melanoma detection in this study was lower (32.5%) than the reported specificity of clinical examination (88%) and dermoscopy (91%) by referral centers/experts,^13^ but all lesions in this study were preselected for biopsy by dermatologist-investigators, and thus, these values are not directly comparable as well. The AUROC is an additional measure of diagnostic accuracy that was similar in this study for the device and dermatologist-investigators in the detection of melanoma (AUROC 0.758 vs 0.747; *P* = .829).^14^

Our findings suggest that the ESS device is likely best used when there is clinical suspicion for melanoma, with a negative ESS result interpreted as highly unlikely to be melanoma or SDN. ESS device use may allow for prioritization of individuals with high-risk skin lesions as classified by the device. The device may also yield benefit as an adjunct in teledermatology settings as dermatologists have been found to have a sensitivity of 67.7% and specificity of 38.6% for melanoma detection with dermoscopic and clinical images alone.^15^ The ESS device is handheld, portable, requires minimal training, and does not require additional materials/reagents.

The ESS device may be particularly useful in areas that are cosmetically-sensitive or at risk for poor wound healing and in patients with numerous atypical-appearing lesions.^16^ Although the ESS device had a PPV of 16.0% and specificity of 32.5%, the device correctly classified 32.5% of benign lesions that were biopsied by dermatologist-investigators. The PPV of 16.0% equates to a NNB of 6.3, compared to a NNB of 10 for the dermatologist-investigators. The device yielded a negative result of “Monitor” for 2 histopathologic melanomas (4.5%), and thus, biopsy is warranted when there is sufficient clinical and/or dermoscopic suspicion for melanoma. Additionally, the ESS device does not account for patient history and similarity to other lesions, which are critical in clinical decision-making.^17^ Device performance differed by lesion size, with a lower specificity for detecting melanoma in lesions ≥6 mm than for lesions <6 mm. This finding could be due to higher variability between the 5 device recordings during examination of larger lesions.

Although the PPV for melanoma detection with a spectral score of 6-10 was 2.5 times higher than those with a spectral score of 1-5, there were histopathologic melanomas in the 1-5 spectral score group. A spectral score of 8-10 exhibited a PPV approaching 50%; such lesions should be triaged quickly to biopsy. Further research is needed to evaluate characteristics of melanomas that received a lower spectral score to improve device performance.

Previously FDA-approved devices for melanoma detection include Nevisense (SciBase AB) and MelaFind (MELA Sciences Inc). The Nevisense system uses similar spectroscopy technology, electrical impedance spectroscopy, to detect melanoma and has a sensitivity of 96.6% (one-sided 95% lower confidence bound of 94.2%) and specificity of 34.4% (95% CI, 32.0% to 36.9%).^17^ MelaFind’s AI multispectral camera system for melanoma detection has a sensitivity of 98.4% (one-sided 95% lower confidence bound of 95.6%) and specificity of 9.5% (95% CI, 6.1% to 12.9%).^18^ Compared to these devices, the ESS device is simple to use, requires minimal training, and provides an immediate result. Additionally, unlike Nevisense and MelaFind, the ESS device was developed for detection of all common skin cancer types, not melanoma specifically.^17^^,^^18^

Other AI technologies for melanoma detection currently lack FDA approval. Moleanalyzer Pro (Fotofinder Systems) uses a convolutional neural network to classify skin lesions using dermoscopic images.^19^ Moleanalyzer Pro has been shown to have a comparable sensitivity (81.6%, 95% CI, 66.6% to 90.8%) and a higher specificity (88.9%, 95% CI, 83.7% to 92.7%) for melanocytic lesions than dermatologists, including lesions in special sites (ie, acral, nails).^19^ However, the time and effort required to take and upload dermoscopic images may limit use of this technology. Publicly available smartphone and web-based dermatology apps using AI diagnostics have an overall sensitivity of 28% (95% CI, 17% to 39%) and a specificity of 81% (95% CI, 71% to 91%).^20^ As these apps are publicly available, use may cause false reassurance or alternatively, increased healthcare utilization.^20^

Limitations include study design, as this study was conducted by dermatologists in high-volume melanoma dermatology clinics with a high cancer prevalence. Device performance in amelanotic melanomas is unknown as the majority of lesions included were pigmented. Lesions in nonaccessible sites (eg, under nails), adjacent to or on scars/areas of past surgical intervention, acral surfaces, mucosal surfaces, or near the eye were not included; device performance in these settings is unknown. All lesions in this study were clinically suspicious for melanoma per board-certified dermatologists and selected for biopsy; therefore, the benign lesions in this study were likely not representative of such lesions in the general population. Due to study design, we lack information about dermatologist-investigators’ false negative rate and biopsy sensitivity as well as device performance in lesions not selected for biopsy by dermatologist-investigators. This may limit the generalizability of the sensitivity and specificity in this study with respect to the general population, who may have lower skin cancer baseline risk and may present with lower risk skin lesions.

## Conclusion

The handheld, noninvasive ESS device reported herein exhibited high sensitivity and negative predictive value for melanoma detection and had AUROC comparable to that of dermatologist-investigators. Coupled with skin examination findings, this device may aid clinicians in melanoma detection. The device may be particularly useful as a triage tool for high-risk lesions, for lesions in cosmetically sensitive locations or areas with poor wound healing, in patients with numerous atypical-appearing lesions, and as an adjunctive tool for teledermatology.

## Conflicts of interest

Drs Tepedino, McNiff, Fung, and Hartman were all provided funding for their participation in the study. Mr Gianacas is a paid consultant for DermaSensor, Inc. Dr Grant-Kels is an uncompensated member of the Advisory Board for DermaSensor, Inc.
